# Cases Bearing on Some Doubtful Points in the History of Syphilis

**Published:** 1877-03-20

**Authors:** Osgood Mason

**Affiliations:** New York


### CASES BEARING ON SOME DOUBTFUL POINTS IN THE HISTORY OF SYPHILIS.

By Dr OSGOOD MASON, M. D., New York.

The *Record* of October 21st ult., under the head of "Progress of Medical Science," gives the result of certain observations by M. Diday concerning the syphilitic infection of the mother through the fœtus.

It is well known that amongst specialists and teachers in this branch of medical science the possibility of such infection has, to say the least, been looked upon with doubt and disfavor. M. Diday's investigations in this direction, however, have led him to the conclusion that "the child begotten by a syphilitic father, without actual lesions, may infect the mother at any period of its intra-uterine life," either as ovule, embryo, or fœtus. At the time the item above referred to came to my notice, I had under observation the following case:

Mrs. A., æt. thirty years, and three years married, was at the time of her marriage an unusually strong, robust, and healthy looking woman. Soon after marriage she became pregnant, and miscarried at four and a half months, with a fœtus at about three months' development, on which occasion I first attended her. After some months she again became pregnant, and was delivered at term. I did not see her during this pregnancy nor at the confinement. I know, however, that she had a most miserable recovery, which was impeded by most distressing and obstinate mammary abscesses, and followed by general ill-health. I was called to see the child when it was six months old ; it was then moribund, and died the following morning of exhaustion, partly from diarrhœa, but especially from a number of large blebs or superficial abscesses situated mostly about the head, and containing an abundance of thin, sanious pus.

I saw no more of the family for several months, until in September last I was called to attend Mrs. A. for dysentery already of a week's duration. It yielded somewhat to rest and opium treatment ; it would not, however, remain under control, but gradually changed its character to a thin, dark diarrhœal discharge, always worse at night, which continued, notwithstanding my best endeavors, for full five weeks, during which time I also ascertained that she was two months or more pregnant.

At this time (October 17th) Mr. A., the husband, was taken ill with symptoms of meningitis. He had a pulse of 64, partial paralysis of the right side, and stupor, from which he was aroused with considerable difficulty, when he would just recognize those about him, answer some simple question, and immediately fall off again to sleep.

Mr. A. had been my patient for many years, and his previous history has a peculiar bearing upon his own case as well as that of his wife. Twelve years ago he contracted syphilis, was thoroughly treated and apparently cured. Two years later the disease again made its appearance in the form of nocturnal headaches and gummy tumors about the elbows. He was then treated with large doses of iodide of potash, and made a prompt recovery, though treatmeut was continued for some months.

He was at that time a married man, and was the father of healthy children. After recovery from this attack, his wife again became pregnant, and was delivered of a healthy child, which is still alive and apparently free from syphilitic taint.

Soon after this the wife died of tubercular disease, and a second marriage was contracted, the results of which have already been given. They sum up as follows:

1st. A miscarriage, attended with symptoms of a suspicious character.

2d. A syphilitic child, followed by unusually severe and obstinate mamary abscesses and failing health.

2d. A pregnancy which in the second month is accompanied by syphilitic symptoms on the part of the mother, at the same time that symptoms reappear in a dangerous form in the father.

The illness of Mr. A. at once cleared up the whole case, and the diarrhœa, which had resisted five weeks of thorough ordinary treatment, with the advantage of rest and care, yielded in six days to two grains of blue mass, given four times a day, while going about and attending upon her husband.

Mr. A. also rapidly recovered under the use of large doses of iodide of potash and mercurial inunctions. By large doses of pot. iod. is meant a drachm every four hours, or, perhaps still better, half that quantity every two hours, until decided iodism, as indicated by the breath, is produced, and the same repeated at proper intervals afterwards.

I am also kindly permitted by my friend Dr. Loomis, of this city, to refer to the following case, which came under his observation, and which, if possible, is still more definite and cogent in its bearing.

Eighteen years ago a young man, then recently treated by the Doctor for syphilis, both primary and secondary, came again for advice concerning marriage, and was advised to delay. Soon after, however, he took counsel of his inclinations and married a young and perfectly healthy wife. In the course of the next five years two healthy children were born, both of whom are still alive and apparently free from any syphilitic taint. The next pregnancy terminated in a miscarriage, with a syphilitic fœtus. At this time the husband had a return of secondary symptoms, and soon after the wife was attacked with syphilitic rheumatism and sore throat. These yielded to appropriate treatment and pregnancy again occurred, but terminated also in a miscarriage, and was soon after followed by periostitis and suppurative nodes. Treatment again caused a disappearance of the symptoms and return to health, and for several years no returning symptoms manifested themselves. Two years ago, however, she was again attacked, this time the disease assuming the form of pachymeningitis, with most alarming symptoms,—paralysis, stupor, and even decided coma, continuing several days, and followed by delirium and dementia. This case also yielded to the large doses of iodide of potash, and resulted in a slow and only fair recovery.

An important, and, in fact, the main question in both these cases is, how did the mothers contract syphilis—by primary infection, or through pregnancy? And in answer I would submit the following considerations:

1st. Both the fathers were treated by the physicians respectively reporting the cases, and were known to have had syphilis in both its primary and secondary forms, thus rendering a second primary infection, to say the least, very improbable, if not impossible. Besides, each father was under the care of these physicians from the time of the first infection until the the time when syphilis was developed in the mother, and no second infection occurred.

2d. The character and social position of both mothers, well known to their respective physicians, are sufficient guarantee that infection did not take place from persons other than their own husbands; and, besides, no such primary esion is known or suspected by their medical attendants to have existed.

3d. Syphilitic symptoms did not occur until after pregnancy, but were developed during pregnancy or very soon after; leaving, as it seems to me, no fair conclusion but that the infection was a direct result of pregnancy.

It is worthy of remark in both these cases, that decided syphilitic symptoms first showed themselves in the mother at or about the same time that symptoms of returning disease manifested themselves in the father.

As a last important and curious consideration, I would call attention to the fact that healthy children, still living under observation, and still free from any manifestation of syphilitic disease, were begotten by both these fathers after they had developed and been treated for secondary syphilis; and then, subsequently, without any new infection, they have begotten children through whom, and during whose intra-uterine life syphilis was communicated to the mothers.—*Medical Record.*

